# A multicenter study of ICU resource utilization in pediatric, adolescent and young adult patients post CAR-T therapy

**DOI:** 10.3389/fonc.2022.1022901

**Published:** 2022-10-24

**Authors:** Dristhi Ragoonanan, Saleh Bhar, Gopi Mohan, Fernando Beltramo, Sajad J. Khazal, Caitlin Hurley, Clark Andersen, Steven Margossian, Sattva S. Neelapu, Elizabeth Shpall, Cristina Gutierrez, Priti Tewari, Basirat Shoberu, Aimee Talleur, David McCall, Cesar Nunez, Branko Cuglievan, Francesco Paolo Tambaro, Demetrios Petropoulos, Hisham Abdel-Azim, Kris M. Mahadeo

**Affiliations:** ^1^ Department of Pediatrics, Stem Cell Transplantation and Cellular Therapy, University of Texas MD Anderson Cancer Center, Houston, TX, United States; ^2^ Texas Children’s Hospital, Baylor College of Medicine, Houston, TX, United States; ^3^ Division of Pediatric Hematology-Oncology, Boston Children’s Hospital, Boston, MA, United States; ^4^ Children’s Hospital Los Angeles, University of Southern California Keck School of Medicine, Los Angeles, CA, United States; ^5^ Department of Bone Marrow Transplantation and Cellular Therapy, St Jude Children’s Research Hospital, Memphis, TN, United States; ^6^ Department of Biostatistics, Division of Basic Sciences, The University of Texas MD Anderson Cancer Center, Houston, TX, United States; ^7^ Department of Lymphoma and Myeloma, The University of Texas MD Anderson Cancer Center, Houston, TX, United States; ^8^ Department of Stem Cell Transplantation and Cellular Therapy, The University of Texas MD Anderson Cancer Center, Houston, TX, United States; ^9^ Department of Critical Care, The University of Texas MD Anderson Cancer Center, Houston, TX, United States; ^10^ Department of Pediatrics, The University of Texas MD Anderson Cancer Center, Houston, TX, United States; ^11^ Pediatric Stem Cell Transplantation and Cell Therapy Program, UOC SIT-TMO AORN Santobono-Pausilipon, Napoli, Italy; ^12^ Division of Transplant and Cell Therapy, Loma Linda University Cancer Center, Loma Linda, CA, United States

**Keywords:** Immunotherapy, CAR (chimeric antigen receptor) T-cell therapy, pediatric cancer, AYA (adolescents and young adults), Resource utilisation

## Abstract

Tisagenlecleucel is associated with remarkable outcomes in treating patients up to the age of 25 years with refractory B-cell acute lymphoblastic leukemia (ALL). Yet, due to unique and potentially life-threatening complications, access remains limited to higher-resource and certified centers. Reports of inequity and related disparities in care are emerging. In this multicenter study of ALL patients admitted for anti-leukemia therapy, who required pediatric intensive care (ICU) support (n = 205), patients receiving tisagenlecleucel (n = 39) were compared to those receiving conventional chemotherapy (n = 166). The median time to ICU transfer was 6 (0–43) versus 1 (0–116) days, respectively (p < 0.0001). There was no difference in the use of vasopressor, ionotropic, sedating, and/or paralytic agents between groups, but use of dexamethasone was higher among tisagenlecleucel patients. Patients receiving tisagenlecleucel were more likely to have cardiorespiratory toxicity (p = 0.0002), but there were no differences in diagnostic interventions between both groups and/or differences in ICU length of stay and/or overall hospital survival. Toxicities associated with tisagenlecleucel are generally reversible, and our findings suggest that resource utilization once admitted to the ICU may be similar among patients with ALL receiving tisagenlecleucel versus conventional chemotherapy. As centers consider improved access to care and the feasibility of tisagenlecleucel certification, our study may inform strategic planning.

## Introduction

Therapeutic strategies for patients with relapsed or refractory (R/R) B-cell acute lymphoblastic leukemia (ALL) may differ based on disease characteristics, cooperative group recommendations, and resource availability. (1) Chimeric antigen receptor T-cell (CAR-T) therapy is a promising strategy for patients with R/R ALL. Tisagenlecleucel has demonstrated impressive minimal residual disease negative remission rates of 81% at 3 months (2). Yet, CAR-T therapy is associated with unique and potentially life-threatening toxicities including cytokine release syndrome (CRS) and immune effector cell-associated neurotoxicity syndrome (ICANS) (3). Up to 40% of patients receiving tisagenlecleucel may require transfer to the intensive care unit (ICU) (2). While the availability of tisagenlecleucel has been limited to certified centers with adequate training and resources to deliver this therapy safely and effectively, emerging reports of disparities in therapy suggest that wider availability may be indicated (4, 5). We hypothesized that among patients admitted for anti-ALL therapy who require ICU support, ICU resource utilization and outcomes would not differ among patients receiving tisagenlecleucel versus those who did not.

## Methods

This study was reviewed by the Pediatric Acute Lung Injury and Sepsis Investigators (PALISI) Network, Hematopoietic Cellular Therapy-Cancer Immunotherapy Subgroup and approved by the institutional review board (IRB) at each participating PALISI Network sites (n = 5). We conducted a retrospective analysis of patients up to age 25 years who received tisagenlecleucel for ALL and required admission to the ICU between 1 November 2017 and 1 June 2020. Patients with ALL receiving conventional chemotherapy admitted to the ICU during this period were used as comparators. CRS and ICANS toxicities were graded as per the American Society for Transplantation and Cellular Therapy (ASTCT) (6). Patients with incomplete medical records and those receiving CAR-T therapy other than tisagenlecleucel for ALL were excluded.

Data extracted from the electronic medical record included demographics, reason for ICU admission, incidence and grading of CRS and ICANS, pediatric sequential organ failure assessment (pSOFA) score (7), resource utilization including imaging, procedures and medications, ICU and overall hospital length of stay (LOS), and mortality.

Patients’ demographic and clinical characteristics were summarized as median and range for continuous variables and as frequency and percentage for categorical variables and compared between patient groups admitted to the ICU who did and did not receive tisagenlecleucel using t-test, Mann–Whitney test, negative binomial regression for continuous variables, or Fisher’s exact test or chi-square test for discrete variables, as appropriate. ICU survival and hospital survival were summarized by Kaplan–Meier methods, with differences between patient groups assessed by the log-rank test. Statistical analyses were performed using R statistical software (8). Statistical significance was set at a p-value of <0.05.

## Results

Of the patients with ALL admitted to the ICU (n = 205), 39 patients (19.0%) received tisagenlecleucel and 166 (81.0%) did not. Patient characteristics, resource utilization, and outcomes are summarized in **Table 1**. Patients undergoing CAR-T therapy were older as they underwent conventional chemotherapy prior. The most common indication for ICU admission in the non-CAR-T therapy group was respiratory failure requiring mechanical ventilation (n = 82; 49.4%), whereas hypotensive shock (associated with CRS) was the most common indication in the CAR-T therapy group (n = 22; 56.4%). Non-CAR-T therapy patients were more likely to be admitted to the ICU for hyperleukocytosis (p = 0.001). The median time to ICU admission from day of hospital admission was shorter in the conventional chemotherapy group at 1 day (0–116 days) versus 6 days (0–43 days) in the CAR-T therapy group, respectively (p < 0.0001).

**Table 1:** Characteristics, resource utilization, and clinical outcomes of patients with acute lymphoblastic leukemia admitted to the intensive care unit.

		CAR-T therapy n = 39	Non-CAR-T therapy n = 166	P value
**Age (years)**		13 (1.5-25)	11 (0.3-25)	0.047
**Gender**	**Men**	16 (41.0%)	111 (66.9%)	0.003
	**Women**	23 (59.0%)	55 (33.1%)	
**Prior hematopoietic cell transplantation**	**Yes**	8 (20.5%)	41 (24.7%)	0.68
	**No**	31 (79.5%)	125 (75.3%)
**Days from CAR-T therapy to ICU admission**		6 (0-43)	—	<0.0001
**Days from hospital admission to ICU admission**		—	1 (0-116)
**pSOFA score on admission to the ICU**		6 (1-12)	6 (0-17)	0.67
**Max pSOFA score during ICU admission**		8 (1-18)	9 (1-23)	0.81
**Reason for ICU admission**	**Respiratory failure**	17 (43.6%)	82 (49.4%)	0.59
	**Shock**	22 (56.4%)	72 (43.4%)	0.16
	**Altered mental status**	9 (23.1%)	22 (13.3%)	0.14
	**Renal failure**	3 (7.7%)	18 (10.8%)	0.77
	**Seizures**	0 (0%)	9 (5.4%)	0.21
	**Hyperleukocytosis**	0 (0%)	31 (18.7%)	0.001
**Medications**	**Vasopressors**	23 (59.0%)	73 (44.0%)	0.11
	**Inotropes**	2 (5.1%)	17 (10.2%)	0.54
	**Sedatives**	14 (35.9%)	82 (49.4%)	0.15
	**Paralytics**	5 (12.8%)	27 (16.3%)	0.81
	**Dexamethasone**	19 (48.7%)	2 (1.2%)	<.0001
**Max CRS score**	**1**	3 (7.7%)	
	**2**	9 (23.1%)	
	**3**	14 (35.9%)	
	**4**	13 (33.3%)	
**Max ICANS score**	**0**	17 (43.6%)	
	**1**	3 (7.7%)	
	**2**	10 (25.6%)	
	**3**	6 (15.4%)	
	**4**	3 (7.7%)	
**Evidence of liver dysfunction^1^**		30 (76.9%)	123 (74.1%)	0.84
**No. of patients requiring paracentesis** **Median no. of paracentesis performed**		2 (5.1%) 0 (0-3)	6 (3.6%) 0 (0-10)	0.65
**Evidence of cardiotoxicity^2^**		31 (79.5%)	87 (52.4%)	0.002
**No. of patients requiring ECHOs** **Median no. of ECHOs performed**		26 (66.7%) 1 (0-4)	119 (71.7%) 1 (0-8)	0.56
**Transesophageal echocardiogram**		0 (0%)	4 (2.4%)	1.0
**No. of patients requiring EKGs** **Median no. of EKGs performed**		27 (69.2%) 1 (0-16)	135 (81.3%) 1 (0-19)	0.12
**No. of patients requiring pericardiocentesis**		0 (0%)	1 (0.6%)	1.0
**No. of patients requiring cardiac catheterization**		0 (0%)	2 (1.2%)	1.0
**Evidence of respiratory toxicity^3^**		28 (71.8%)	82 (49.4%)	0.013
**Evidence of cardiac and/or respiratory toxicity**		38 (97.4%)	119 (71.7%)	0.0002
**No. of patients requiring invasive mechanical ventilation**		6 (15.4%)	58 (34.9%)	0.02
**No. of patients requiring CPAP** **Median duration of CPAP (days)**		5 (12.8%) 0 (0-5)	13 (7.8%) 0 (0-27)	0.35
**No. of patients requiring BiPAP** **Median duration of BiPAP (days)**		10 (25.6%) 0 (0-8)	52 (31.3%) 0 (0-68)	0.56
**No. of patients requiring HFNC** **Median duration of HFNC (days)**		18 (46.2%) 0 (0-37)	53 (31.9%) 0 (0-31)	0.1
**No. of patients requiring chest X-rays** **Median no. of chest X-rays performed**		33 (84.6%) 4 (0-60)	155 (93.4%) 4 (0-83)	0.1
**No. of patients requiring bronchoscopy** **Median no. of bronchoscopies performed**		1 (2.6%) 0 (0-1)	24 (14.5%) 0 (0-2)	0.05
**No. of patients requiring tracheostomy** **Median no. of tracheostomies performed**		0 (0.0%) 0	2 (1.2%) 0 (0-1)	1.0
**No. of patients requiring thoracentesis** **Median no. of thoracentesis performed**		1 (2.6%) 0 (0-1)	11 (6.6%) 0 (0-2)	0.47
**No. of patients requiring CRRT**		6 (15.4%)	30 (18.1%)	0.82
**No. of patients requiring CT brain** **Median no. of CT brain performed**		13 (33.3%) 0 (0-2)	50 (30.1%) 0 (0-5)	0.7
**No. of patients requiring MRI brain** **Median no. of MRI brain performed**		7 (17.9%) 0 (0-3)	35 (21.1%) 0 (0-7)	0.83
**No. of patients requiring EEG** **Median no.of EEG performed**		12 (30.8%) 0 (0-17)	35(21.1%) 0 (0-17)	0.21
**No. of patients requiring LP** **Median no. of LPs performed**		5 (12.8%) 0 (0-7)	61 (36.7%) 0 (0-5)	0.004
**ICU LOS (days)**		6 (2-55)	7.5 (1-125)	0.22
**Hospital LOS (days)**		28 (5-150)	21 (1-183)	0.019
**Death during ICU admission**		6 (15.4%)	45 (27.1%)	0.03
**Death during hospital admission**		8 (20.5%)	48 (28.9%)	0.33

^1^Defined as new-onset CTCAE ≥Grade 3 transaminitis, coagulopathy, or hepatomegaly.

^2^Defined as new-onset cardiomyopathy, arrhythmia, tachycardia, hypotension, or hypotensive shock.

^3^Defined as hypoxia requiring any oxygen supplementation or respiratory failure.

CAR-T, chimeric antigen receptor T cell; ICU, intensive care unit; pSOFA, pediatric sequential organ failure assessment; CRS, cytokine release syndrome; ICANS, immune effector cell-associated neurotoxicity syndrome; ECHO, echocardiogram; EKG, electrocardiogram; CPAP, continuous positive airway pressure; BiPAP, bilevel positive airway pressure; HFNC, high flow nasal cannula; CRRT, continuous renal replacement therapy; CT, computerized tomography; MRI, magnetic resonance imaging; EEG, electroencephalogram; LP, lumbar puncture; LOS, length of stay.

All CAR-T therapy patients admitted to the ICU had CRS and/or ICANS. Seventeen patients (43.6%) developed CRS only, and 22 (56.4%) patients had concurrent CRS and ICANS. Twenty-seven (69.2%) patients developed a maximum CRS score of ≥Grade 3, eight of which had concurrent ICANS Grade ≥ 3. Given the high incidence of CRS, patients in the CAR-T therapy group were more likely to have evidence of cardiac toxicity (defined as new-onset cardiomyopathy, arrhythmia, tachycardia, or hypotension/shock) compared with the non-CAR-T therapy group (79.5% *vs*. 52.4%; p = 0.002). Likewise, respiratory toxicity (defined as hypoxia requiring oxygen supplementation or respiratory failure) was higher in the CAR-T versus the non-CAR-T therapy group, respectively (71.8% *vs*. 49.4%; p = 0.013). Despite the higher incidence of respiratory toxicity in the CAR-T therapy group, invasive mechanical ventilation (15.4% *vs*. 34.9%; p value = 0.02) and bronchoscopies (2.6% *vs*. 14.5%; p = 0.05) were lower in the CAR- therapy *vs*. the non-CAR-T therapy groups, respectively.

There were no significant differences between groups in the use of procedures including paracentesis, pericardiocentesis, cardiac catheterization, thoracentesis, tracheostomy, or continuous renal replacement therapy. A higher proportion of patients in the non-CAR-T therapy group underwent lumbar puncture (p = 0.004) to facilitate the administration of intrathecal chemotherapy. There was no significant difference in the use of vasopressors, inotropes, sedatives, or paralytics between both groups. The use of dexamethasone was significantly higher in the CAR-T therapy group for the treatment of CRS/ICANS (48.7% *vs*. 1.2%; p < 0.0001).

There was no significant difference in the number of imaging investigations between groups, including transesophageal echocardiogram, echocardiogram (ECHO), electrocardiogram (EKG), chest X-ray, magnetic resonance imaging (MRI), and computer tomography (CT) of the brain and electroencephalogram (EEG).

**Figure 1:**
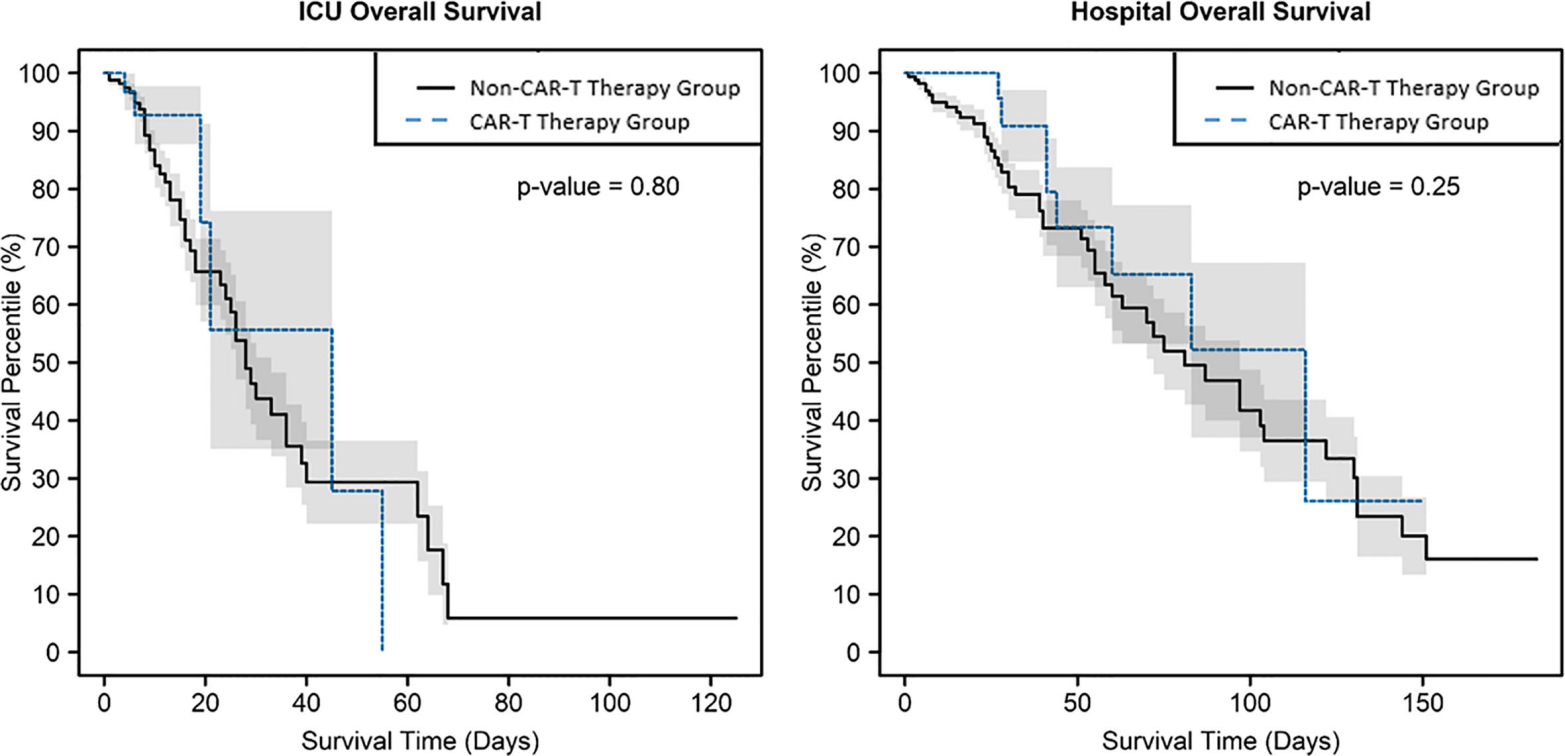
Kaplan–Meier survival curves, which account for mortality over time, with shaded +/- standard error, showed no evidence of difference by CAR-T therapy group in intensive care unit mortality or hospital mortality, with p = 0.80 and 0.25, respectively.

Median ICU length of stay (LOS) was similar in the CAR-T and non-CAR-T therapy groups, respectively (6 (2–55) versus 7.5 (1–125) days; p = 0.22). Overall hospital LOS was longer in the CAR-T *vs*. the non-CAR-T therapy group (28 (5–150) *vs*. 21 (1–183) days; p = 0.019), which may be associated with a longer preceding time to ICU admission in the CAR-T therapy group. The pSOFA score, which is a measure of organ dysfunction with higher scores on ICU admission being associated with higher in-hospital mortality (7), was comparable between the CAR-T therapy *vs*. non-CAR-T therapy groups (6 (1–12) *vs*. 6 (0–17), respectively; p = 0.67). ICU mortality was higher in the non-CAR-T therapy than the CAR-T therapy group (27.1% *vs*. 15.4%; p = 0.03), although the difference in overall hospital mortality was not significant (28.9% *vs*. 20.5%; p = 0.33). Neither ICU nor hospital mortality differed significantly between CAR-T groups per log-rank test (**Figure 1**).

## Discussion

CAR-T therapy has revolutionized the therapeutic landscape for patients with R/R ALL who previously had limited treatment options. While its short-term benefits are well established, given its lack of durable response in 50% of patients at 12 months and with an estimated lifetime cost of $667,000, tisagenlecleucel is currently the most expensive oncological therapy whose long-term benefit remains to be established (2, 9). In 2018, however, the institute for clinical and economic review estimated that the cost-effectiveness of tisagenlecleucel fell within commonly cited thresholds for cost-effective oncology drugs of $50,000 to $150,000/QALY over a lifetime with 10.34 life years and 9.28 QALYs gained with tisagenlecleucel compared with 2.43 life years and 2.10 QALYs gained with a conventional chemotherapy-based regimen (9).

To our knowledge, this is the first multicenter study to explore resource utilization in pediatric patients admitted to the ICU for CAR-T therapy-related complications. Previous reports suggest that up to 40% of patients receiving tisagenlecleucel may require ICU support (2). Our study was limited to outcomes of patients admitted to the ICU. As expertise grows, however, ICU admission rates for patients receiving CAR-T therapy may decline as many toxicities may be managed without ICU intervention.

In this study, overall hospital LOS and time to ICU admission were longer in patients undergoing CAR-T therapy as they were all admitted for lymphodepletion at least 6 days pre-infusion as per standard of care. Furthermore, patients in the non-CAR-T group were more likely to be admitted due to the acute nature of complications secondary to their disease course and/or treatment such as septic shock or leukocytosis at initial diagnosis. During their admission, overall resource utilization appears comparable in patients with ALL receiving CAR-T therapy and conventional chemotherapy. Additionally, CAR-T therapy and non-CAR-T therapy patients appear to have similar organ dysfunction and expected risk of hospital mortality upon ICU admission (p-SOFA), although our study did not analyze the effect of poor prognostic factors or cause of mortality. CAR-T therapy patients, however, appear to require less invasive mechanical ventilatory support and may demonstrate superior outcomes, which is likely reflective of the potentially reversible toxicities of CRS and ICANS when recognized and treated promptly (10, 11).

Overall, while the administration of CAR-T therapy is associated with increased upfront costs, resource utilization in these patients requiring critical care is comparable with ALL patients undergoing conventional chemotherapy. Given its remarkable remission rates, CAR-T therapy is, therefore, an excellent therapeutic strategy. As more centers introduce CAR-T therapy, rigorous protocols for clinical monitoring and prompt toxicity management available at certified centers may mitigate ICU admissions and support needs (11). Longer-term studies, however, are needed to fully understand the critical care needs of patients undergoing CAR-T therapy.

## Data availability statement

The raw data supporting the conclusions of this article will be made available by the authors, without undue reservation.

## Ethics statement

Ethical review and approval was not required for the study of human participants in accordance with the local legislation and institutional requirements. Written informed consent from the patients OR patients legal guardian/next of kin was not required to participate in this study in accordance with the national legislation and the institutional requirements.

## Author contributions

DR, CR, and KM designed the study and wrote the protocol and the manuscript. DR, SB, GM, FB, SK, CH, CA, BS, SM, SN, ES, CG, AT, PT, DM, CN, BC, FPT, DP, HA, and KM assisted with data collection and review. CA led the biostatistical analysis for this study. All authors contributed to the article and approved the submitted version.

## Acknowledgments

We thank our patients and nursing unit colleagues. We acknowledge the members of the PALISI Network HCT-Cancer Immunotherapy Subgroup for the scientific review of our study.

## Conflict of interest

KM is the site PI for Atara Biotherapeutics, Jazz Pharma, Allovir, and BMS. CG has served in the Advisory Board for Legend Biotech & Janssen.

The remaining authors declare that the research was conducted in the absence of any commercial or financial relationships that could be construed as a potential conflict of interest.

The handling editor declared a past collaboration with author KM.

## Publisher’s note

All claims expressed in this article are solely those of the authors and do not necessarily represent those of their affiliated organizations, or those of the publisher, the editors and the reviewers. Any product that may be evaluated in this article, or claim that may be made by its manufacturer, is not guaranteed or endorsed by the publisher.
